# Kinetic Effects of Transferrin-Conjugated Gold Nanoparticles on the Antioxidant Glutathione-Thioredoxin Pathway

**DOI:** 10.3390/antiox12081617

**Published:** 2023-08-15

**Authors:** Sonia Sebastian, Manuela Klingler Hoffmann, Douglas Howard, Clifford Young, Jenni Washington, Harald Unterweger, Christoph Alexiou, Tyron Turnbull, Richard D’Andrea, Peter Hoffmann, Ivan Kempson

**Affiliations:** 1 Future Industries Institute, University of South Australia, Adelaide, SA 5095, Australia; sonia.sebastian@mymail.unisa.edu.au (S.S.); douglas.howard@mymail.unisa.edu.au (D.H.); tyron.turnbull@unisa.edu.au (T.T.); 2 Clinical Health Sciences, University of South Australia, Adelaide, SA 5000, Australia; manuela.klingler-hoffmann@unisa.edu.au (M.K.H.); clifford.young@unisa.edu.au (C.Y.); peter.hoffmann@unisa.edu.au (P.H.); 3 Mass Spectrometry & Proteomics Group, Clinical Health Sciences, University of South Australia, Adelaide, SA 5000, Australia; 4 Department of Otorhinolaryngology, Head and Neck Surgery, Section of Experimental Oncology and Nanomedicine (SEON), Else Kröner-Fresenius-Stiftung Professorship, Universitätsklinikum Erlangen, 91054 Erlangen, Germany; harald.unterweger@uk-erlangen.de (H.U.); christoph.alexiou@uk-erlangen.de (C.A.); 5 Centre for Cancer Biology, University of South Australia, Adelaide, SA 5000, Australia; richard.dandrea@unisa.edu.au

**Keywords:** antioxidant, nanoparticle, reactive oxygen species, glutathione-thioredoxin, proteomics, TEM

## Abstract

Nanoparticle-based therapeutics are being clinically translated for treating cancer. Even when thought to be biocompatible, nanoparticles are being increasingly identified as altering cell regulation and homeostasis. Antioxidant pathways are important for maintaining cell redox homeostasis and play important roles by maintaining ROS levels within tolerable ranges. Here, we sought to understand how a model of a relatively inert nanoparticle without any therapeutic agent itself could antagonize a cancer cell lines’ antioxidant mechanism. A label-free protein expression approach was used to assess the glutathione-thioredoxin antioxidative pathway in a prostate cancer cell line (PC-3) after exposure to gold nanoparticles conjugated with a targeting moiety (transferrin). The impact of the nanoparticles was also corroborated through morphological analysis with TEM and classification of pro-apoptotic cells by way of the sub-G0/G1 population via the cell cycle and annexin V apoptosis assay. After a two-hour exposure to nanoparticles, major proteins associated with the glutathione-thioredoxin antioxidant pathway were downregulated. However, this response was acute, and in terms of protein expression, cells quickly recovered within 24 h once nanoparticle exposure ceased. The impact on PRDX-family proteins appears as the most influential factor in how these nanoparticles induced an oxidative stress response in the PC-3 cells. An apparent adaptive response was observed if exposure to nanoparticles continued. Acute exposure was observed to have a detrimental effect on cell viability compared to continuously exposed cells. Nanoparticle effects on cell regulation likely provide a compounding therapeutic advantage under some circumstances, in addition to the action of any cytotoxic agents; however, any therapeutic advantage offered by nanoparticles themselves with regard to vulnerabilities specific to the glutathione-thioredoxin antioxidative pathway is highly temporal.

## 1. Introduction

The earliest evidence of the tumorigenic role of antioxidant pathways was obtained in a clinical trial in 1996, wherein a population with a high risk of developing lung cancer with severe smoking habits were administered vitamin A and β carotene dietary supplements to curb their cancer [1]. The incidence rate of lung cancer actually increased to 28% for the active treatment group compared to the placebo group. The beta carotene levels in all patients in the active treatment group were 12 times more than what is considered as the normal level in people. Higher levels of β carotene may have caused an imbalance with other antioxidant compounds in the body, which are important for the redox mechanisms and probably many other cellular pathways which can be harmful for the body [2,3]. Recent studies have further established the role of antioxidant pathways in cancer cell survival and metastasis [4,5]. For example, Sayin et al. demonstrated that lung cancer cells in mice that were given high doses of antioxidants grew more rapidly and were more likely to spread to other parts of the body than in mice that were not given antioxidants [6]. The researchers hypothesized that the antioxidants may protect cancer cells from damage caused by reactive oxygen species (ROS), allowing them to grow and divide more easily [6]. Another study aimed to investigate the impacts of antioxidants on the development and metastasis of melanoma in mice showed that treatment with antioxidants increased the number of metastases, and the antioxidant-induced metastases were more invasive and aggressive than those observed in control mice [7]. The antioxidant treatment caused changes in the expression of genes involved in metastasis and immune regulation, which was associated with increased tumor invasiveness and immune suppression. The study indicates that the use of antioxidant supplements may promote the metastasis of melanoma and other cancers [7].

Reactive Oxygen Species (ROS), including hydroxyl radicals (OH∙), superoxide anions (O2∙^−^), and hydrogen peroxide (H_2_O_2_) [8,9] are highly reactive molecules that regulate various metabolic pathways in cellular organelles like mitochondria, peroxisomes, and the endoplasmic reticulum [10,11]. Redox homeostasis involves the generation and elimination of the ROS maintaining the intracellular balance of these species [12,13].

Cancer cells are highly metabolic and often display constant production of intracellular ROS greater than normal cells [14,15] with upregulated antioxidative systems to avoid cellular damage and cell death [16,17]. The glutathione-thioredoxin pathway plays an important role in protecting cancer cells from excessive ROS levels generated during cell metabolism [18,19]. Studies have shown that these antioxidant systems are highly upregulated and expressed in aggressive forms of metastatic cancers which may associate with poorer prognosis [20,21]. Maintaining the intracellular balance of ROS is critical for cellular homeostasis and differences in ROS levels can cause different biological responses within the cells. ROS sustain cellular proliferation and metastasis in cancer cells [14]; however, beyond a threshold, it can lead to cancer cell apoptosis or other forms of cell death [22]. The glutathione-thioredoxin pathways are one of the most well researched endogenous antioxidant systems in cancer cells [18,23]. 

**Figure 1:**
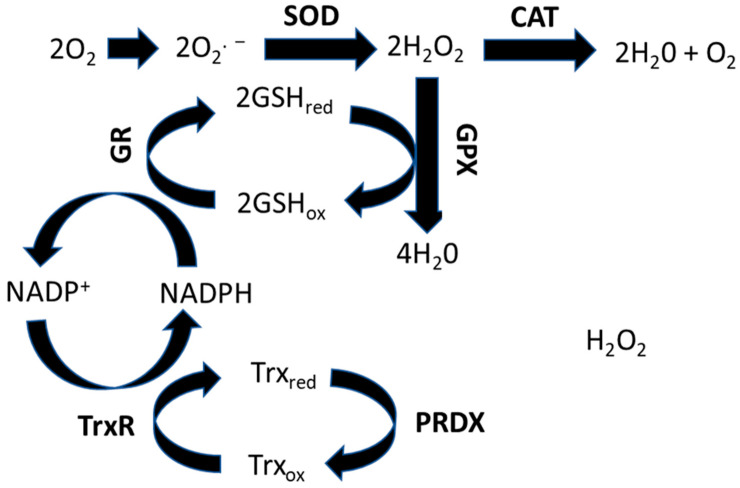
Schematic representation of the glutathione-thioredoxin pathway for maintaining the cell redox homeostasis.

The glutathione-thioredoxin systems are the most abundant endogenous antioxidant systems [24,25] (Figure 1) and a key compound in this system is glutathione (GSH), with its concentration inside cells being much greater than any other antioxidant proteins [26,27]. GSH is synthesized via a two-step process, in which first glutamate and cysteine are ligated, followed by catalysis and the addition of glycine by glutathione synthetase (GSS) [28,29]. 

Glutathione peroxidase (GPX) and glutathione reductase (GR) are crucial for a fully functional GSH system [30,31]. Glutathione peroxidase 1 (GPX1) is the most common gene isoform of glutathione peroxidase in mammalian cells with both in vitro and in vivo studies confirming its role in counteracting ROS-induced stress [32,33,34,35]. GPX continuously converts H_2_O_2_ to H_2_O by consuming GSH. This results in the conversion from the reduced form of GSH to its oxidized form GSH_ox_. The glutathione reductase (GR) reverts the GSH_ox_ back to GSH_red_ by using NADPH. This pathway is the most significant pathway involved in metabolizing H_2_O_2_ in most cells [36,37]. 

Thioredoxin (Trx) systems are thiol-dependent and consist of thioredoxin (Trx), thioredoxin reductase (TrxR), and NADPH [38]. Trx functions by either neutralizing ROS via recycling peroxiredoxins (PRDX) or by reducing proteins in oxidized forms, which oxidizes Trx. After proteins are brought to their reduced state, the oxidized Trx is then reduced back to its normal active state by the utilization of NADPH by TrxR [39,40]. Peroxiredoxins (PRDX) are a family of proteins that function as protein substrates of Trx1. A total of six PRDX proteins have been identified in mammals, out of which PRDX 1–4 are crucial for reducing H_2_O_2_ [41]. These proteins cycle back to their active forms using Trx. PRDX5 also acts as a substrate for Trx but prefers reducing alkyl hydroperoxides over H_2_O_2_. PRDX6 is actively involved in the reduction of phospholipid hydroperoxides, and functions independently of Trx [42]. They use GSH to process their catalytic reaction. TrxR, a selenoprotein and an important contributor in the antioxidant system, has two main forms in mammals, cytosolic TrxR1 and mitochondrial TrxR2 [43]. Both forms contain a flavin adenine dinucleotide (FAD) binding domain, an interface domain, and a NADPH binding domain. 

In a mouse model of breast cancer, inhibiting the glutamate cysteine ligase modifier (GCLM), important for glutathione (GSH) synthesis, diminished malignancy and progression of the tumor [19]. However, blocking GSH synthesis after tumor establishment has failed to reduce the tumor burden and progression [19]. This suggested that inhibiting GSH is effective only before the onset of tumor formation. In addition, the study identified that other antioxidative systems may compensate for loss of GSH, thus protecting the cancer cells from ROS damage. Inhibiting GSH synthesis was found to increase the levels of thioredoxin (Trx), another protein involved in the endogenous antioxidant pathway system. Inhibiting both GSH and Trx had a synergistic inhibition on the tumor growth in vivo [19].

Another way of modulating GSH activity is to inhibit GPX4, a selenoprotein used by GSH as a co-factor to modulate lipid peroxidation in cells during increased oxidative stress. The inhibition of GPX4 using RSL3, a small molecule inhibitor, caused suppression of the mTOR pathway as well as the phosphorylation of the DNA damage response protein nucleophosmin 1 (NPM1), causing ferroptosis in papillary thyroid cancer cells [44]. Concomitant targeting of GSH synthesis and NADPH oxidases (NOX) induced cell death in cancer xenografts harboring oncogenic RAS such as Ras transformed ovarian epithelial cells as well as mutant KRAS-harboring pancreatic and colon cancer cells of mouse and human origins [45].

Formulations comprising metal nanoparticles to target cancer cells are increasingly prevalent in translational research [46,47]. Metal nanoparticles can create and promote imbalance in redox homeostasis by generating more ROS inside cells via Haber-Weiss and Fenton-type reactions and also by depleting the ROS scavenging pathways by overwhelming the cellular antioxidant systems and causing oxidative stress [48]. Studies using cerium oxide (CeO) nanoparticles, for example, have been driven by mimicking both superoxide dismutase and catalase activities, thus exhibiting redox-modulatory activities in cells. Surprisingly, the catalase mimicking activity of the CeO nanoparticles significantly decreased at acidic pH, suggesting that they could induce cytotoxicity and oxidative stress in the acidic microenvironment of highly glycolytic tumors [49]. However, at neutral pH of normal tissues, the CeO nanoparticles induced antioxidative or protective effects [49]. Consistent with the above study, both the in vivo and in vitro studies of CeO against melanoma cells demonstrated an anticancer effect of CeO by inducing apoptosis in cancer cells in vitro and reducing the tumor size and growth in animal models in vivo without affecting nearby normal tissues [50]. 

Nanoparticles have been shown to enter sub cellular organelles and cause increased ROS levels by triggering the electron transport chain (ETC) and activating NADPH-dependent oxidases [51]. Based on this, super-paramagnetic iron oxide (SPION) nanoparticles endocytosed and localized in lysosomes were shown to dissociate and release iron ions, which then translocated to the mitochondria where they targeted the ETC systems like the Fe-S clusters and heme, thus producing O_2_∙^−^ species [52]. In a similar context, manganese oxide nanoparticles were shown to increase the ROS levels inside the mitochondria by altering the electron transport chain complexes I and III and reduced the activity of complexes II and IV, which resulted in deteriorating mitochondrial activity [53]. Titanium dioxide and iron oxide nanoparticles have the ability to activate NADPH oxidases, thus generating more O_2_∙^−^ [54,55]. Similarly, gold ions exert toxicity and oxidative stress [56]. Au(I) complexes with chelated diphosphines can inhibit TrxR and GPX activity with an increase in intracellular ROS at 24 h in lymphoma cells [57]. Au-based nanoparticles however are generally considered inert and biocompatible leading to extensive prevalence in nanomedical research [58,59].

While nanoparticles can alter redox status in cells, these have generally been with nanoparticles that can dissolve or release ions intracellularly and result in direct chemical imbalances. Little information exists on how the mere physical presence of nanoparticles may influence cell biochemistry and redox homeostasis. Furthermore, little is known about the temporal effects after acute or continuous exposure to nanoparticles on intracellular antioxidant systems. In light of the number of nanomedicine formulations comprised of metal nanoparticles undergoing preclinical development, it is important to understand the impacts from nanoparticles themselves, in addition to any cytotoxic ligands or functionality. In this study, temporal impact of transferrin-conjugated gold nanoparticles (Au-Tf) on the glutathione-thioredoxin pathway have been explored under acute and chronic exposure conditions. Gold metal is generally considered as being biocompatible and biologically inert and, hence, little influence was expected due to potential dissolution of metal ions from the nanoparticles. The gold nanoparticles were conjugated with PEG and transferrin as previous studies from our group were able to show that conjugation with transferrin improved cellular up-take up to one order of magnitude greater than coating with PEG alone [60]. Transferrin receptors are often over expressed on cancer cells, providing a targeting opportunity through transferrin functionalization [61,62]. Changes in protein regulation associated with the glutathione-thioredoxin pathway were analysed using proteomics. Proteomics gives a better understanding of whether a particular protein is actually synthesized in the cells or not, which can be irrespective of gene copy number. The observations made from proteomics were corroborated with TEM structural and compositional analysis along with quantification of pre-apoptotic fractions. These findings are not only useful for understanding how nanoparticles sensitize cells to therapies like radiotherapy or chemotherapy, but also can be used for generally understanding how nanoparticles can interfere with cellular function to understand their toxicity in normal tissues.

## 2. Materials and Methods

### 2.1. Synthesis and Characterisation of Transferrin-Conjugated Gold (Au-Tf) Nanoparticles

The gold (Au) nanoparticles (~15–18 nm) were synthesized via the established Turkevich method [63]. Then 1 mL of Au nanoparticle solution was spun down at 15,000 rpm, 4 °C for 15 min and the pellet was resuspended with 750 µL of cold Milli Q water. To the solution, 62 µL of o-(2-carboxyethyl)-o′-(2-mercaptoethyl) heptaethylene glycol (PEG, Sigma Aldrich 672688-250 MG, St. Louis, MO, USA) MW~500, and 338 µL of poly (ethylene glycol) 2-mercaptoethyl ether acetic acid (PEG, Sigma Aldrich 757845-500 MG, St. Louis, MO, USA) MW~5000 were added and sonicated for 5 min at room temperature. The solution mixture was then refrigerated overnight for 12–14 h for PEG conjugation onto the nanoparticles. After 12–14 h, the solution mixture was spun down at 15,000 rpm, 4 °C for 15 min and resuspended in cold Milli Q water to obtain the Au-PEG solution. 

The Au-PEG solution was centrifuged at 15,000 rpm, 4 °C to obtain the Au-PEG nanoparticle pellet. To the pellet, 300 µL of N-(3-Dimethylaminopropyl)-N′-ethyl carbodiimide hydrochloride (EDC, Sigma Aldrich E6383-5 G, St. Louis, MO, USA): N-Hydroxysuccinimide (NHS, Sigma Aldrich 130672-250 G, St. Louis, MO, USA) solution (76.7 mg EDC and 11.5 mg NHS in 1 mL of Milli Q water) and 600 µL of Milli Q water were added. The solution was sonicated for 10 min at room temperature after which it was centrifuged at 12,000 rpm, 20 °C for 30 min. To the pellet, 40 µL of human holo transferrin (hTf, Sigma Aldrich T0665-100 MG, St. Louis, MO, USA) and 400 µL of phosphate buffer saline (PBS, Thermofisher Scientific, 14190144, Waltham, MA, USA, Gibco, Grand Island, NY, USA) were added. After sonication for 10 min, the mixture was incubated for 2 h at room temperature to allow for Tf conjugation onto the Au-PEG nanoparticles. After incubation period, the nanoparticles were centrifuged at 12,000 rpm, 20 °C for 30 min. The pellet was redispersed in sterile PBS to obtain transferrin-conjugated gold nanoparticles (Au-Tf). The transmission electron micrograph of the nanoparticles was done using JEOL JEM-2100F-HR transmission electron microscope (JEOL group company, Scientific/Metrology Instruments, Akishima, Tokyo, Japan) with a constant voltage of 200 kV. The UV-VIS absorbance of Au, Au-PEG, and Au-Tf nanoparticles were measured to confirm the successful transferrin conjugation of the nanoparticles. Dynamic light scattering (DLS) was used to characterize the size of nanoparticles during each synthesis step. For stability measurements using dynamic light scattering measurements, samples were diluted to 2 nM with RPMI 1640 medium. The hydrodynamic size distributions were determined with a Malvern Nano ZS (Malvern Panalytical, Spectris scientific, London, UK) in backscattering mode (173°) at 25 °C. 

### 2.2. Cell Culture Preparation for the Exposure of PC-3 Cells Exposed to Au-Tf Nanoparticles 

The PC-3 (ATCC CRL-1435™) cells were sub-cultured into two six well plates with a seeding density of ~500,000 cells in each well and supplemented with media enriched with 10% fetal bovine serum (FBS, Thermofisher Scientific Gibco A4736201, Waltham, MA, USA, Gibco, Grand Island, NY, USA) and 1% penicillin/streptomycin antibiotic (P/S, Thermofisher Scientific Gibco 15070063, Waltham, MA, USA, Gibco, Grand Island, NY, USA). After 24 h in incubator at 37 °C and 5% CO_2_, nine of the wells were washed twice with serum-free media and treated with 6 nM Au-Tf nanoparticles dispersed in serum-free media itself. This concentration was chosen based on a previous study from our group confirming the biological changes in cells at this concentration [64].

### 2.3. Exposure Conditions Used for Uptake Analysis, Proteomics, Transmission Electron Microscopy (TEM), and Cell Cycle Study of the Nanoparticles in Cells

**Figure 2:**
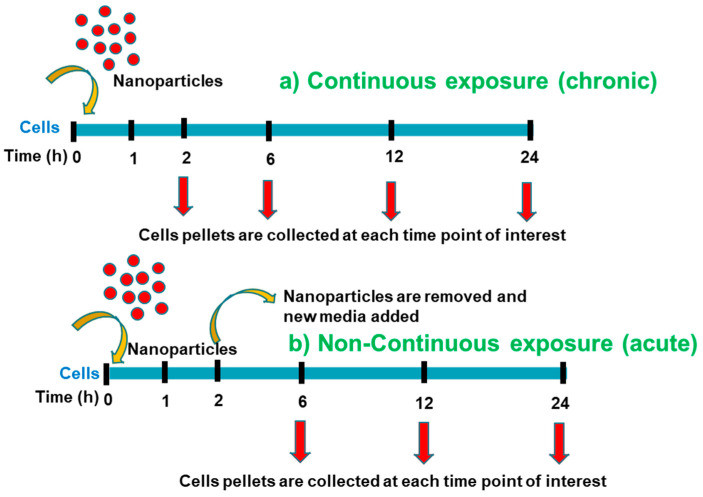
(**a**) Experimental procedure for the continuous exposure with nanoparticles; (**b**) Experimental procedure for the non-continuous exposure with nanoparticle.

Two different exposure conditions were considered, continuous exposure (chronic) and the non-continuous exposure (acute) of Au-Tf (6 nM) nanoparticles, as shown in Figure 2. For the continuous exposure, nanoparticles were continuously exposed to cells without removing the nanoparticles and replenishing the media. For the non-continuous exposure, cells were exposed to nanoparticles for 2 h, after which they were removed, and the cells were replenished with fresh serum-free media containing no nanoparticles. These cells were then grown for an additional 4–22 h. 

### 2.4. Reactive Oxygen Species Analysis

ROS were measured using the fluorescent dye, 2′,7′-Dichlorofluorescein (DCFDA, Sigma Aldrich 410217-1 G, St. Louis, MO, USA) in cell samples. PC-3 cells were plated in a 48 well plate at a concentration of ~1 × 10^4^ cells using RPMI-1640 media not containing phenol red and left to attach overnight. Cells were then washed with PBS and 100 µL of Au-Tf nanoparticle was added and incubated for 2 h. After 2 h, the wells containing cells were washed with PBS. Then 100 µL of PBS was added to the wells and 30 µL of 10 µM DCFDA was added. Well plates were protected from light and analysed using a FLUOstar Optima Plate Reader (BMG Labtech, Ortenberg, Germany) at fluorescence excitation of 495 nm and emission of 525 nm for analysis of dichlorofluorescein (DCF).

### 2.5. Sample Processing for the ICP-MS Analysis for the Uptake of Nanoparticles in Each Exposure Condition in PC-3 Cells

For performing the ICP-MS of PC-3 cells with nanoparticles, the cells were collected at different time points by trypsinization, followed by neutralizing with RPMI media. These samples were transferred to fresh 15 mL tubes, weighed and properly labelled. These were then freeze dried in *Modyulo* freeze dryer (Thermo Electron, Waltham, MA, USA) for three days to remove all liquid and only obtain the powdered sample. The tubes with powder samples were weighed post freeze drying to obtain the total weight of the powder samples.

For obtaining consistent results, sub samples were equally weighed into 1.5 mL eppendorf tubes from parent samples and digested with 3:1 Hydrochloric acid (HCl, ChemSupplyAustralia, RP1106-G2.5 L, Gillman, SA, Australia): Nitric acid (ChemSupplyAustralia VL1137C-G2.5 L, Gillman, SA, Australia) (Aqua regia) overnight. The caps of the eppendorf were left loose so that there was enough room for the effervescence to escape and avoid popping of samples. The digested samples were diluted 10 times with thiourea diluent (prepared in the lab).

For the standard calibration, 0.1 ppb, 0.5 ppb, 1 ppb, 5 ppb, 10 ppb, 20 ppb, 50 ppb, 100 ppb, 500 ppb, 1000 ppb, 2 ppm, 5 ppm, 8 ppm, and 10 ppm Au in thiourea diluents were prepared. The standard calibrants and samples were run through an Agilent 8900 Triple Quad ICP-MS instrument (Agilent Life sciences, Santa Clara, CA, USA).

### 2.6. Downstream Processing Steps for Cell Sample Processing for Non-Labelled Proteomics

**Cell lysis**: The cell media from the 6 well plates were removed and washed twice gently with PBS. Around 500 µL of RIPA buffer [65] was added to each well incubated in ice. The cells were scraped from the wells using a scraper to detach them from the bottom of the wells. Using a pipette, the cell lysate was mixed up and down many times to form a homogenous lysate. After 5 min, the lysate was transferred to a 1.5 mL microcentrifuge tube and spun down at 14,000× *g* for 15 min to remove cell debris and separate the protein. The supernatant was transferred to a new Lo-Bind 1.5 mL (Eppendorf, 0030108442, Tokyo, Japan) tube and stored at −20 °C until further processing. For each of the protein samples, ice cold acetone (Sigma Aldrich 179124-1 L, St. Louis, MO, USA) four times the volume of the starting protein supernatant was added. The tube was gently inverted at least three times. The samples were then placed at −20 °C freezer overnight for best results. 

**Acetone precipitation, protein digestion, and sample clean up**: The centrifuge was precooled to −9 °C before spinning the samples down at 20,000× *g* for 10 min. The acetone supernatant was removed without disturbing the pellet, and the samples were spun again for 5 min to remove excess acetone. The protein pellets were washed twice with 200 µL of fresh ice-cold acetone before being left to air dry over ice. The pellets were then dissolved in 8 M urea (Sigma Aldrich U5378-500 G, St. Louis, MO, USA) in 50 mM ammonium bicarbonate (AmBiC, Sigma Aldrich 09830-1 KG, St. Louis, MO, USA) (usually the volume is ~10 µL). 

The protein samples were sonicated on ice for 2–3 min and then incubated with dithiothreitol (DTT, Sigma Aldrich 43815-1 G, St. Louis, MO, USA) at room temperature for 1 h. Chloroacetamide (CAA, Sigma Aldrich C0267-500 G, St. Louis, MO, USA) was added to the samples, and they were incubated in the dark at room temperature for 30 min. The samples were then diluted with 50 mM AmBiC to avoid trypsin inhibition due to high urea concentration. Trypsin was added to the samples, and they were incubated at 37 °C for 18 h. After 18 h, the samples were spun down gently to collect any condensation. To the samples, 10% formic acid (FA, ChemSupplyAustralia AC1085005P, Gillman, SA, Australia) was added to achieve a final concentration of 1% volume. The final pH was at 2–3 to ensure maximum binding of peptides during sample cleanup. C18 zip tips (Merck Millipore, C5737, Burlington, MA, USA) were used for sample cleanup. The zip tips were equilibrated with acetonitrile (ACN, Merck P.N. K50182327 818, Rahway, NJ, USA) and 0.1% formic acid (FA, ChemSupplyAustralia AC1085005P, Gillman, SA, Australia) before sample loading. The protein digest was loaded onto the zip tips and washed with 0.1% formic acid. The sample was eluted with 50% acetonitrile in 0.1% formic acid before drying down and storing at −20 °C until loaded into the Orbitrap Exploris™ 480 Mass Spectrometer instrument (Thermo Fisher Scientific, Bremen, Germany) for analysis.

LC-MS analysis was conducted on an EASY-nLC 1200 system (Thermo Scientific, Thermo Fisher Scientific, Bremen, Germany) coupled to an Orbitrap Exploris 480 mass spectrometer (Thermo Scientific, Thermo Fisher Scientific, Bremen, Germany). Approximately 1 ug of peptide sample was loaded onto a 50 °C heated C18 fused silica column (75 um inner diameter, 360 um outer diameter, 25 cm length) at a flow rate of 600 nLmin^−1^. The column was in-house packed with 1.9 um ReproSil-Pur 120 C18-AQ particles (Dr Maisch). Chromatography was performed over a 70 min linear gradient (3–20% acetonitrile in 0.1% formic acid) at a flow rate of 300 nLmin^−1^. Two compensation voltages (−50 and −70 V) were alternately applied from a FAIMS Pro interface (Thermo Scientific, Thermo Fisher Scientific, Bremen, Germany) to adjust the entry of ionised peptides into the mass spectrometer. MS scans (*m*/*z* 300 to 1500) were acquired at resolution 60,000 (m/z 200) in positive ion mode for data-dependent acquisition. MS/MS scans were measured at resolution 15,000 after multiply charged peptide precursors (minimum intensity 10,000) were sequentially fragmented with higher energy collision dissociation (HCD) at 27.5% normalized collision energy. A dynamic exclusion period of 40 s was specified, and cycle times were restricted to 1.5 s.

### 2.7. Processing of Raw Data for the Non-Labelled Proteomics

The raw data from the LC-MS instrument was processed through Maxquant version 1.6.17.0. The proteingroups.txt file obtained from Maxquant was then analysed using Perseus software version 1.6.15.0. The raw data excel sheets generated from Perseus are included in the Supplementary Information (Table S1). The core proteins involved in the glutathione-thioredoxin pathways were run through STRING proteomics software (Version number 12.0) to obtain the string network analysis for each of the exposure conditions. The mass spectrometry proteomics data have been deposited to the ProteomeXchange Consortium via the PRIDE [66] partner repository with the dataset identifier PXD039433″.

### 2.8. Transmission Electron Microscope (TEM) Biological Sample Preparation to Study Au-Tf Nanoparticle Uptake in PC-3 Cells

Cell pellets were washed with Dulbecco’s phosphate buffer saline (DPBS, Thermofisher Scientific Gibco 14190144) and fixed using 2.5% glutaraldehyde (GA, ProSciTech, EMS16216, Kirwan, QLD, Australia) and 2% paraformaldehyde (PF, ProSciTech C004, Kirwan, QLD, Australia) overnight at room temperature. After removing the fixative, the cell pellet was then washed with DPBS twice. The cell pellet was subjected to osmification using 1% osmium tetroxide (OsO_4_, ProSciTech EMS19190, Kirwan, QLD, Australia) and 1.5% potassium ferrocyanide (K₃ [Fe (CN)₆], Sigma Aldrich 60279-250 G, St. Louis, MO, USA) solution for 1 h. The mixture was removed, and the cell pellet was washed twice with DPBS. 

The cell pellet was dehydrated with Ethanol (EtOH, ChemSupplyAustralia scientific EA043-2.5L-J, Gillman, SA, Australia) gradient (30%, 50%, 70%, 90%), each for 15 min and then removed. The pellet was finally dehydrated in 100% EtOH twice, each for 15 min and then removed. The cell pellet was slowly introduced to Epon 812 resin (ProSciTech C038, Kirwan, QLD, Australia) for embedding. At first, a ratio of 2:1 propylene oxide (PO, Sigma Aldrich 110205-500 ML, St. Louis, MO, USA): Epon 812 resin was added to the cell pellets and incubated for 1 h at room temperature. Then, a ratio of 1:1 of the same was added and incubated for 1 h. Finally, a ratio of 1:2 of the same was added and incubated for 1 h. The cell pellet was then introduced to pure Epon 812 resin and left for 1 h. 

After removing the mixture, fresh Epon 812 resin was introduced and left overnight. The next day, the cell pellet was covered in fresh epon resin, and the resin was dried in the oven for two days at 60 °C. The embedded resin samples were sectioned using an ultramicrotome for obtaining ultrathin sections (~70–90 nm) of the sample. The thin sections were collected onto a 200-mesh copper/palladium grids and dried at room temperature. The grids supporting the sections were post stained using gadolinium acetate (Ted Pella 19485, Redding, CA, USA) for 15 min and Fahmy lead citrate for 5–8 min. For Fahmy lead citrate stain, 50 mL of Milli Q water was boiled to which one pellet of sodium hydroxide (NaOH, Sigma Aldrich S5881-500 G, St. Louis, MO, USA) and 0.25 g of lead citrate (ProSciTech C073, Kirwan, QLD, Australia) were added. After drying at room temperature, the grids were imaged using Tecnai Biotwin TEM (FEI company, Hillsboro, OR, USA) at Adelaide Microscopy (University of Adelaide, SA, Australia).

### 2.9. Cell Cycle Analysis of Au-Tf Nanoparticle-Treated PC-3 Cells Were Treated with 6 nM Au-Tf Nanoparticles for 2 h, 24 h Continuous, and 24 h Non-Continuous Periods

PC-3 cells were treated with Au-Tf nanoparticles as described above. A non-treated cell sample was kept as a control. The cells were collected by trypsinization, and the cell pellets were washed with ice-cold 1X PBS (DPBS, Thermofisher Scientific Gibco 14190144, Waltham, MA, USA, Gibco, Grand Island, NY, USA) twice before fixing them with 70% (*v*/*v*) ethanol (EtOH, ChemSupplyAustralia scientific EA043-2.5L-J, Gillman, SA, Australia) overnight. After overnight fixation at 4 °C, the cells were washed again with 1X PBS twice. The cells were then stained with DAPI (Thermofisher Scientific, D1306, Waltham, MA, USA) for 10 min at room temperature before flow cytometry analysis was performed. The samples were analysed using a BD LSRFortessa X-20 Cell Analyser (BD Biosciences, Becton Dickinson, Franklin lakes, NJ, USA). The cell cycle graphs were plotted using the flow cytometry analysis software Floreada.io. 

### 2.10. Detection of Early Apoptotic Cell Populations Using Annexin V Early Apoptosis Detection Kit

PC-3 cells were treated with Au-Tf nanoparticles as described above. Non-treated PC-3 cells were also cultured as controls. At the experiment time, one flask of the control cells was detached and exposed to 2% paraformaldehyde to obtain a population of dead cells. Another flask was treated with 6% H_2_O_2_ for 30 min to obtain early an apoptotic cell population. A control dual dye sample was prepared by mixing the control cells, dead cells, and early apoptotic cells. 

For the apoptosis assay, an annexin V-FITC apoptosis detection kit (APOAF-20TST) was purchased from Sigma Aldrich. Since the reagents need to be stored at 4–8 °C, they were allowed to warm up to room temperature before using them for the assay. The binding buffer was prepared by dissolving 1 mL of the 10X binding buffer solution provided, with 9 mL of Milli Q water. After the treatment with nanoparticles, the treated PC-3 cells were trypsinized and then neutralized with media. After centrifugation for 5 min at 500× *g*, the supernatants were removed and washed with 1X PBS. This step was repeated once again. After removing the supernatant, 1X binding buffer was added to all the treated samples as well as the dual control sample. To each of the samples, 5 µL of annexin (FITC-A) and 10 µL of propidium iodide (PI) were added and incubated for 10 min. The samples were analysed using a BD LSRFortessa X-20 Cell Analyser. Data were plotted using the flow cytometry analysis software Floreada.io.

### 2.11. Cell Viability Assay of Nanoparticle-Treated and Control Cell Populations Using Trypan Blue

The number of viable cells was counted after trypan blue staining for two samples taken from each condition run in triplicate. Data were normalized to the number of cells counted for each time point with no nanoparticle exposure. Nanoparticle-exposed cells experienced additional washing and were normalized to data for cells which were subjected to an additional wash to account for cells being lost due to the physical disruption from washing. Standard error was determined for each condition and propagated by the addition of the relative error through the normalization calculation.

## 3. Results

### 3.1. Nanoparticle Characterisation, Uptake, and ROS Analysis

**Figure 3:**
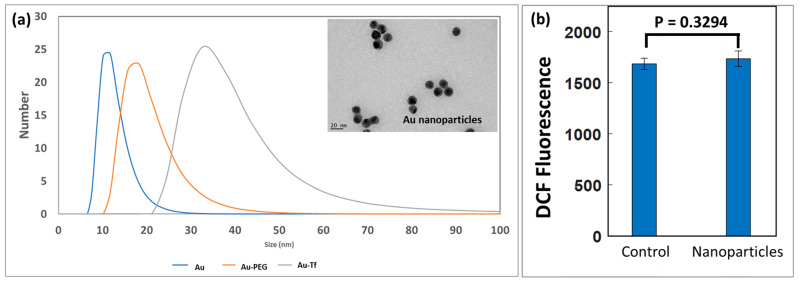
(**a**) DLS data showing number versus size distribution for Au, Au-PEG, and Au-Tf nanoparticles. Transmission electron microscopy (inset) indicated an Au nanoparticle diameter of ~18 nm; (**b**) ROS data measured by the DCFCDA assay indicated a slight ROS increase in cells after 2 h exposure to nanoparticles, although it was not significant.

Transmission electron microscope (TEM) images showed the Au nanoparticles were ~18 nm in size, as shown in Figure 3a. The UV-VIS absorbance measurement of Au, Au-PEG, and Au-Tf nanoparticles confirmed the successful conjugation of PEG and transferrin onto the nanoparticles (Supplementary Information Figure S1). The DLS data showed the number versus size distribution for the Au, Au-PEG, and Au-Tf nanoparticles. The size of the Au-PEG and Au-Tf increased during conjugation of the PEG and transferrin, respectively, which were observed as a shift to the right in the size distribution (Figure 3a). The Au-Tf nanoparticles were also found to be stable in culture media based on DLS revealing no aggregation (Figure S2). A minor size decrease at the beginning of the measurement was observed. This was due to the measurement commencing as soon as practicable without waiting for temperature equilibrium. The dispersion, taken from refrigeration, was initially colder and affected the light scattering phenomena used for inferring particle size. The particle size remains constant once the temperature equilibrium should have been reached.

ROS are highly regulated by cells and a non-significant difference in DCF intensity was measured for cells exposed to Au-Tf nanoparticles (*p* = 0.3294). (Figure 3b). ICP-MS analysis of the uptake of Au-Tf in PC-3 cells for the different exposure conditions showed an average uptake of ~10 pg of nanoparticles per cell for 2 h, ~11 pg per cell for 24 h continuous, and ~7 pg per cell for 24 h non-continuous exposure; however, uptake is highly heterogenous between individual cells [60,67].

### 3.2. Effect of Au-Tf Nanoparticles on the Differential Protein Expresson of Proteins Involved in the Glutathione-Thioredoxin Pathway of PC-3 Cells

After co-culturing cells with nanoparticles for 2 h, 24 h, or 2 h and then having nanoparticles removed for a further 22 h (24 h non-continuous), the PC-3 cells were analysed for the differential expression of the antioxidant pathway proteins. Biological replicates were lysed, digested with trypsin, and analysed using label-free quantitative proteomics. Proteomics complements genomics by representing actual protein synthesis, which does not always correlate with gene copy number. Each sample was run in technical replicate. The raw data from Maxquant was processed via Perseus software version 1.6.15.0 and two sample T-tests were run to obtain the log *p* values and log fold change values of proteins for each of the conditions with respect to the controls. From the list obtained, proteins of interest that belong to the glutathione-thioredoxin pathway were selectively chosen and compiled, as shown in Table 1.

**Figure 4:**
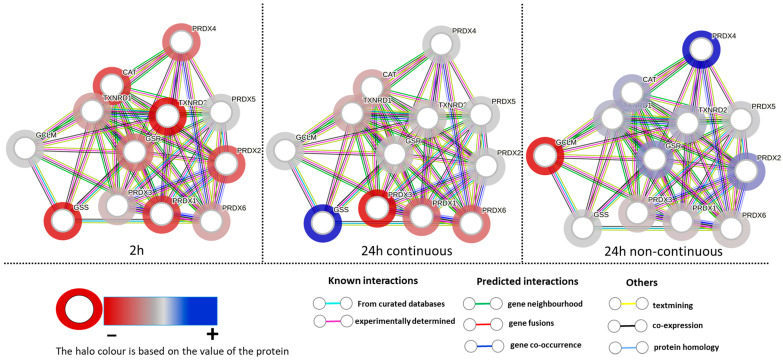
String network analysis for 2 h, 24 h continuous, and 24 h non-continuous exposure showing the interactions between each protein.

Table 1 shows that at 2 h exposure, all detected proteins were downregulated with glutathione reductase (GR) and glutathione peroxidase (GPX) proteins showing significance. Both of these proteins are crucial for the proper functioning of the glutathione thioredoxin system. Hence, it can be interpreted that the glutathione thioredoxin pathway is hindered at 2 h exposure. At 24 h continuous exposure, all detected proteins show probability of downregulation except for the GSS protein. Both peroxiredoxin 1 (PRDX 1) and peroxiredoxin 3 (PRDX 3) proteins were significant and their overexpression in prostate cancer has been proposed to be linked to cancer initiation and progression [41,68]. Peroxiredoxin 3 (PRDX 3) overexpression has been demonstrated to counteract senescence and promote tumor survival in prostate cancer [69]. The peroxiredoxin proteins help in detoxifying peroxides and peroxynitrite derivatives in cells [70]. In 24 h continuous exposure, two of the peroxiredoxin proteins were significantly downregulated and, therefore, the glutathione thioredoxin pathway could be affected to some extent in this condition. At 24 h non-continuous exposure, most proteins were measured to be upregulated, while five other proteins showed a probability of being downregulated. Among the upregulated proteins, the peroxiredoxin 4 (PRDX 4) protein was significantly upregulated and has been demonstrated to play a critical role in prostate cancer cell growth and radioresistance [71]. While many proteins when considered individually are not significantly different, pathway analysis assesses the inter-relation and dependance of each protein within a pathway to determine their collective effect. These observations are visualized by the STRING analysis network shown in Figure 4. 

### 3.3. Effect of Au-Tf Nanoparticles on the Morphology of PC-3 Cells

**Figure 5:**
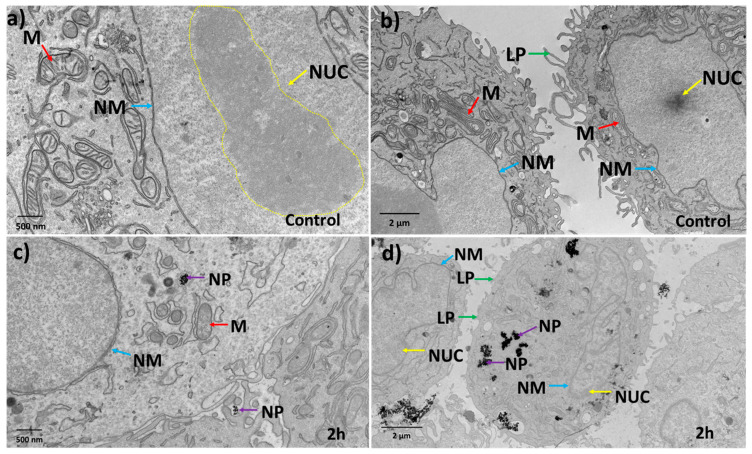
Morphological analysis of cells shows structural changes induced by nanoparticle exposure. (**a**,**b**) Representative control cells without nanoparticle treatment show intact nuclear membranes with nuclear pores well-arranged and with well-defined nucleolus. The lamellopodium structures are well-defined and elongated due to the metastatic nature of the PC-3 cells. (**c**,**d**) 2 h nanoparticle-treated cells show nanoparticles enclosed in vesicular structures and the integrity of the cell nuclear membrane has deteriorated. Chromatin structures have dispersed and moved to the nuclear membrane. Lamellopodium had less integrity and structure. Arrows and abbreviations indicate nuclear membrane (**NM**-blue), nucleolus/chromatin (**NUC**-yellow), lamellopodium structrues (**LP**-green), mitochondria (**M**-red), nanoparticles (**NP**-purple).

**Figure 6:**
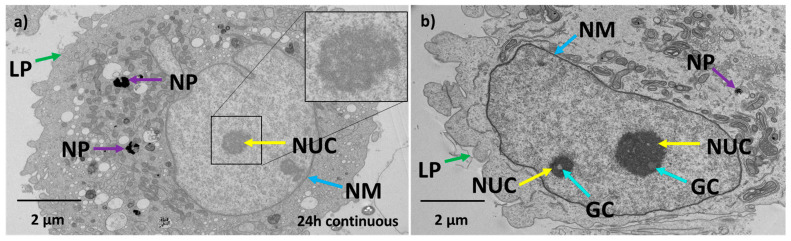
Nanoparticles continue to change cell morphology with continuing exposure, but they can recover. (**a**) TEM image of 24 h continuous exposure of PC-3 cells with Au-Tf nanoparticles. (**b**) TEM image of 24 h non-continuous exposure of PC-3 cells with Au-Tf nanoparticles; Arrows and abbreviations indicate nuclear membrane (**NM**-blue), nucleolus (**NUC**-yellow), lamellopodium structures (**LP**-green), nanoparticles (**NP**-purple), granular compartments (**GC**-turquoise).

Transmission electron microscopy (TEM) has been indispensable for studying the ultrastructural morphological changes occurring within cells. Fine details of intracellular structures can be visualized and monitored for changes to understand the effect of nanomedicines administered on the health and survival of cells [72,73] linked to oxidative stress. TEM images were used to study the effect of nanoparticles on the morphological attributes of the PC-3 cells for all exposure conditions (representative images given in Figure 5 and Figure 6). Figure 5a,b shows control cells have intact nuclear membranes with well-arranged pores. The nucleolus was confined in a defined region within the nucleus, and the DNA can be seen as a graded darker structure within the nucleolus. The control cells also have well-defined lamellipodial structures that are crucial for cell migration (green arrows). The mitochondria were observed as elongated structures with intact cristae (blue arrows).

Figure 5c shows that the 2 h-treated cells had the Au-Tf nanoparticles confined in endolysosomes (purple arrow). The nuclear membrane has started to bulge and lose its integrity. No pores were observed on the nuclear membrane. The mitochondria seemed to have lost its elongated form and instead were seen as rounder structures with deformed cristae (blue arrows). From Figure 5d, it was observed that the treated cells appeared to have lost their well-defined nucleolus, suggesting that the chromatin has moved to the sides of the nuclear membrane which were seen as dark gradient structures near the nuclear periphery (yellow arrow). This is a common feature of apoptotic cells and has been widely reported in the literature [74,75].

After 24 h continuous exposure, TEM analysis showed that nanoparticles (purple arrow) were mostly enclosed in endolysosomes, and the nucleus was rounder in shape (Figure 6a) compared to control cells. The nucleolus was observed to be circular and porous, which is a characteristic phenomenon observed for apoptotic cells [76]. The nuclear membrane continued to show poorer definition similar to the 2 h exposure, such as bulging of the nuclear membrane with no visible pores. The lamellipodial structures lost their shape and the cell membranes were blebbing and being pinched off from the cells. For the 24 h non-continuous exposure as shown in Figure 6b, the nuclear membrane was observed to be reformed with an irregular shape nucleus similar to control cells. However, the nucleolus still appeared rounder with granular clusters inside. These granular compartments are less dense structures found inside nuclei of cells that undergo a stress response [77,78]. Observations across multiple sections of ~20 cells confirmed there were fewer nanoparticles within endolysosomes compared to the 2 h and 24 h continuous exposure. The lamellipodial structures were rounder and had lost their normal form.

### 3.4. Effect of Au-Tf Nanoparticles on the subG0/G1 or Apoptotic Cell Population and Cell Viability

Cell cycle analysis is for analyzing the spread of a cell population across the cell cycle and can also be used for identifying pro-apoptotic cell populations represented by the subG0/G1 population [79]. This information is very useful in understanding whether a drug or nanoparticle causes potential toxic effects to a cell. The cell cycle analysis for the 2 h and 24 h continuous treatment showed a small peak corresponding to the subG0/G1 populations that represents cells prone to apoptosis (Supplementary Information Figure S3). The subG0/G1 populations increased from 2 h to 24 h continuous treatment with the Au-Tf nanoparticles. However, at 24 h non-continuous treatment, the sub G0/G1 peak could not be detected.

**Figure 7:**
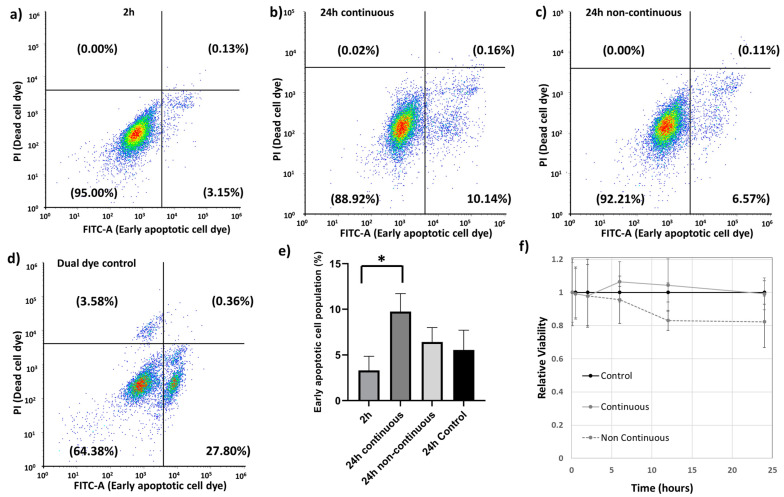
Annexin V early apoptosis measurement assay (**a**) 2 h exposure, (**b**) 24 h continuous exposure, (**c**) 24 h non-continuous exposure, (**d**) dual dye control for determining the different populations, (**e**) comparison of early apoptotic cell populations between different exposure conditions, (**f**) Relative viability for each condition using trypan blue assay; *—Significance *p* < 0.05 (Two paired students *t*-test).

A complementary technique for identifying apoptotic cells is through annexin V staining, which binds to the surface of cells expressing phosphatidylserine, which is an early marker for apoptosis. The assay performed confirmed early apoptotic cell populations in all the treated exposure conditions, as shown in Figure 7a–c. The gating strategy was defined by a dual dye control sample (Figure 7d) comprising a mixture of untreated cells, dead cells, and early apoptotic cell populations. The histograms for both the PI and FITC-A dyes for the dual dye control sample have been provided in the Supplementary Information (Figure S4). The 24 h continuous treatment showed the highest percentage of early apoptotic cells compared to 24 h control and other treatment times, with significant difference between the 2 h and the 24 h continuous exposure (Figure 7e).

A cell viability assay was performed immediately after exposing cells to nanoparticles (5 min) up to 24 h for both the continuous and non-continuous exposure conditions (Figure 7f). For the non-continuous exposure condition, cells were exposed to nanoparticles for 2 h, after which the nanoparticle-containing media was removed and replaced with fresh serum-free media. The non-continuous condition indicates a reduction in cell viability at time points after removal of the nanoparticles. This may be due to these cells experiencing an additional washing step for removing the nanoparticle-containing media and resulting in a loss of a small portion of cells in the process. The apoptotic assay would support this interpretation; however, a reduction in cell proliferation is also possible beyond 2 h.

## 4. Discussion

Advanced metastatic cancers are highly metabolic with higher levels of ROS compared to normal cells [80]. Antioxidative pathways are overexpressed in these cells to cope with the constant production of ROS. One of the obstacles to overcome for targeted therapies focusing on DNA damage with increased ROS production is understanding the tumor microenvironment and redox balance [81]. Inhibiting these pathways likely provides an avenue for favorable treatment outcomes for ROS-targeted therapies. Meanwhile, numerous nanomedicines are being formulated and progressing towards and through clinical translation. However, for many of these formulations, it is not known how the nanoparticles themselves can potentiate any therapeutic strategy. 

Our study focused on studying the effect of transferrin-conjugated gold nanoparticles (Au-Tf) on the antioxidant pathway protein expression, with a specific focus on the glutathione-thioredoxin pathway, cell morphology, and cell cycle of PC-3 metastatic prostate cancer cells for both acute and chronic exposure conditions. The Au-Tf nanoparticles obtained were ~18 nm in size with the average size increasing for Au-PEG and Au-Tf particles during the step-by-step conjugation of PEG and Tf onto the Au nanoparticles. ROS analysis revealed a slight increase in ROS in cells after exposure to nanoparticles, though the increase was not statistically significant compared to non-treated cells. The uptake of Au-Tf nanoparticles measured using ICP-MS showed that the 24 h non-continuous exposure has lower mass of Au-Tf nanoparticles per cell (~7 pg) compared to 24 h continuous exposure (~11 pg), with 2 h exposure having slightly lower mass of Au-Tf nanoparticles (~10 pg) compared to the 24 h continuous exposure. After 2 h exposure of PC-3 cells to Au-Tf nanoparticles, the majority of proteins involved in the glutathione-thioredoxin pathway were found to be downregulated with glutathione reductase and glutathione peroxidase enzymes being significantly downregulated. Glutathione reductase is the key protein responsible for constantly supplying the reduced form of glutathione; one of the most abundant antioxidant molecules present in the majority of cells [82]. The reduced form of glutathione is very important for the cellular maintenance of ROS produced inside cells [26,83]. On the other hand, the antioxidant enzyme glutathione peroxidase is indispensable for free radical scavenging inside cells. This enzyme helps prevent the peroxidation of lipids and helps maintain the redox balance within cells [31,84]. With both these proteins downregulated significantly after 2 h treatment, the antioxidant pathway is altered and possibly the reduction and oxidation of the GSH is not taking place, which might be blocking the pathway from being able to scavenge the free ROS inside these cells. Therefore, the PC-3 cells are vulnerable to oxidative stress and undergo morphological changes signifying the effect of the Au-Tf in these cells, as confirmed by the TEM images (Figure 5c,d). The 2 h-treated PC-3 cells had lost their structural integrity, in contrast to the non-treated control cells (Figure 5a,b). The chromatin had disintegrated and moved to the sides of the nuclear membrane. This is typical in apoptotic cells [85,86]. The cell cycle and apoptosis assay data also support this finding. The cell cycle data of 2 h-treated cells showed a subG0/G1 peak, which corresponds to pro-apoptotic cells or cells that are prone to apoptosis. This was confirmed using the apoptosis assay, wherein it was observed that the 2 h treatment caused a small percentage of the cell population to go into the early apoptotic phase. The PC-3 cells also lost their lamellipodial structures, which are supposed to be elongated rod-like structures that help cancer cells to spread and proliferate [87,88,89,90]. 

Protein expression for 24 h continuous exposure showed that most of the proteins were still slightly downregulated with significant downregulation of the PRDX1 and PRDX3 proteins. For this condition, a slightly greater quantity of nanoparticles was taken up by cells (~11 pg per cell compared to ~10 pg per cell at 2 h). Visualization by TEM also indicated greater numbers of nanoparticles in endolysosomes compared to 2 h exposure. This could be because of chronic exposure for 24 h to nanoparticles, causing a continuous uptake of nanoparticles into the cells greater than any exocytotic processes. Despite this, most proteins analysed were expressed at levels closer to the control cells, suggesting an adaptive response to the stress induced by the nanoparticles. Regardless of any adaptation in protein expression to the nanoparticle exposure at 24 h, a small percent of the cell population was increasingly proapoptotic, represented by an increase in the sub-G0/G1 population, as seen from the cell cycle analysis and also confirmed by the apoptosis assay. This suggests that the significant downregulation of PRDX1 and PRDX3 dominate the ability or inability of the cells to cope with an exogenous nanoparticle stressor. PRDX proteins are usually upregulated in prostate cancer, with PRDX3 and 4 being overexpressed in metastatic prostate cancer, thus helping them cope with oxidative stress and cell survival [68,91]. Hence, downregulation of the PRDX proteins in particular might be affecting the cell survival and their metastatic potential. The TEM image (Figure 6a) is consistent with this observation. It has been proposed that malignant metastatic cancer cells have an irregular nucleus, in contrast to healthy tissue cells [92]. The control PC-3 cells showed an irregular nuclear membrane and shape, which is consistent with this report (Figure 5a). Hence, after 24 h treatment, cells underwent observable morphological change, with the nucleus appearing circular in shape and the nucleolus appearing rounder (Figure 5a). The lamellipodial structures of the cell membrane as well as the nuclear membrane lost their original structure and exhibited blebbing. Cell membrane blebbing is a common feature of cells undergoing apoptosis or any other forms of cell death [93,94]. An increase in transparent vacuolar-like structures within the cytoplasm was also observed, which is also a common feature of cells under stress and has been proposed as a common feature in cell death, though there has not been any quantitative link made [95,96]. The nucleolus appeared less dense compared to the nucleolus of control cells, and also indicates cellular stress and an apoptotic tendency. The continuous exposure showed the relative cell viability for the 24 h continuously exposed cells was almost equal to the control cell population.

In the acute 24 h non-continuous exposure, cells had exocytosed nanoparticles with ~7 pg of nanoparticles per cell remaining internalized. Contrary to the other conditions, many of the proteins were slightly upregulated except for SODC, GCLM, PRDX1, PRDX3, and PRDX6. Out of the upregulated proteins, the PRDX4 proteins were significantly upregulated. An overexpression of PRDX4 has been observed in many cancers including prostate cancer and has been linked with heightened tolerance of cancers to oxidative stress [97,98,99]. There could be two reasons why we see a reversal of the effects of nanoparticle exposure on the glutathione-thioredoxin pathway. Firstly, these cells are exposed to nanoparticles only for a limited time, after which they grow in fresh nanoparticle-free media. During their growth in the media, these cells are likely to exocytose nanoparticles into the media, which reduces the impact from nanoparticles. ICP-MS analysis of the media for the 24 h non-continuous condition, ran in triplicate, confirmed that at 24 h, ~2.4 µg of gold was no longer associated with the cells. Secondly, protein expression has been determined with respect to a control for the same time point and so temporal changes in the control cells can change the apparent protein expression in the treated cells even if no change actually occurred in the treated cells, i.e., an apparent upregulation in the treated cells can be due to a downregulation in the control cells. The TEM analysis (Figure 6b) showed that the cells had morphological similarities to control cells in terms of regaining organelle structures like the endoplasmic reticulum, golgi bodies, and mitochondria. The nuclear membrane appeared to be reformed similar to control cells with less blebbing. However, the cell membrane still looked impaired and the nucleolus small and dense. The appearance of nucleolar cavities called granular compartments was observed (blue arrows in Figure 6b). These nucleolar cavities have been reported to store p53/p21 proteins during the cellular stress response [77,78]. This observation is supported by the apoptosis assay, where a small percentage of cells still in the early apoptosis phase were observed. The relative cell viability (Figure 7f) had also decreased for the different time points in non-continuous exposure compared to the control cell population. However, from the TEM data, the intracellular structures such as the cell organelles appeared more to be like control cells. Intracellular distribution of nanoparticles was still restricted to endolysosomes, although there were fewer of them. Therefore, in 24 h non-continuous exposure, the cells appear to represent non-treated cancer cell-like properties. A similar trend was reported with respect to cell structure and morphology, wherein magnetic iron oxide nanoparticles were shown to stiffen F9 murine embryonal carcinoma cells at ~30 min of exposure [100]. However, at 24 h, the viscoelasticity of the cells reverted back to the same as the control cells [100].

This study observed that Au-Tf nanoparticles inhibit the glutathione-thioredoxin antioxidative pathway. This was most pronounced in short-term acute exposure to nanoparticles. However, downregulation of the specific proteins PRDX1 and 3 correlated a greater proportion of pro-apoptotic or early apoptotic cells during continuous exposure to nanoparticles. The partial adaptive response could be because of a conditioning effect of nanoparticles in cells at longer exposure times, consistent with other studies [100,101]. Our work also indicates that for these conditions and time frames that acute exposure (non-continuous treatment) was potentially more harmful than continuous exposure. This observation is consistent with a prior study exposing fibroblasts to gold nanoparticles and monitoring gene expression [102]. In their work, cells were exposed to a low concentration of nanoparticles for 24 h. If cells were cultured for a further 20 weeks with no nanoparticles, changes in gene expression were reported as being more severe than if the cells continued to be cultured with nanoparticles, in which case they reverted to being more like control cells.

We observed here a kinetic response to nanoparticles in terms of regulation of their antioxidative pathways. This is an important consideration with respect to potential synergistic potentiation of cancer therapeutics. Likewise, nanoparticles could also induce an antagonistic effect compromising therapeutic activity and are highly dependent on timing and pharmacokinetics of the nanoparticles (i.e., short versus long retention times). These observations also have relevance to understanding temporal mechanisms of nanoparticle-induced toxicity in nanoparticle toxicological studies. 

## 5. Conclusions

The effect of transferrin-conjugated Au nanoparticles (Au-Tf) on PC-3 cell’s protein expression, morphology, and pro-apoptotic status was investigated. A time-dependent effect of the nanoparticles was observed. The shortest duration of exposure (2 h) had the greater effect on the antioxidant glutathione-thioredoxin pathways. This effect reversed at 24 h when nanoparticle exposure ceased. If nanoparticle exposure was continuous for 24 h, the cells presented an adaptive response with protein expression partially reverting to be more similar to control cells. Hence, the effects of these nanoparticles in cells were observed to be temporal and most impactful in the acute exposure scenario. Consideration is warranted for how nanoparticles influence nanomedicine-based therapeutics via either synergistic or antagonistic mechanisms by altering cell’s capacity to mediate oxidative stress. These effects, also for nanotoxicology consideration, are dependent on timing, pharmacokinetics, and duration of exposure.

## Figures and Tables

**Table 1:** Change in protein expression after exposure to Au-Tf nanoparticles for different exposure conditions.

Protein Name	Fold Change. 2 h	Fold Change. 24 h Continuous	Fold Change. 24 h Non-Continuous
Glutathione synthase (GSS)	0.32	1.74	ND
Glutathione reductase (GR)	0.6 *	0.96	1.23
Glutathione peroxidase (GPX)	0.5 *	0.75	1
Thioredoxin reductase, mitochondrial (TrxR2)	0.3	0.97	1.1
Thioredoxin reductase, cytoplasmic (TrxR1)	0.75	0.87	1.1
Superoxide dismutase, mitochondrial (SODM)	0.1	0.22	1.23
Superoxide dismutase, cytoplasmic (SODC)	0.35	0.69	0.7
Peroxiredoxin 1,2,3,4, 5 and 6 (PRDX)	0.4, 0.45, 0.87, 0.52, ND, 0.76	0.66 *, ND, 0.45 *, ND, ND, 0.65	0.92, 1.28, 0.91, 2 *, 1, 0.95
Catalase (CAT)	0.4	0.84	1.14
Glutamate--cysteine ligase regulatory subunit (GCLM)	ND	ND	0.22

*—Significance *p* < 0.05 (Two paired students T-test); ND, not detected.

## Data Availability

Data are available from authors on request.

## Supplementary Materials

The following supporting information can be downloaded at: https://www.mdpi.com/article/10.3390/antiox12081617/s1. Figure S1: UV-VIS data showing shift in peak after PEG and transferrin conjugation onto Au nanoparticles; Figure S2: Stability measurement showing the Z-average of Au-Tf nanoparticles in RPMI (nm) with respect to time; Figure S3: Cell cycle data of different treatment conditions with gold nanoparticles showing the sub G0/G1 peaks corresponding to apoptotic cell population; Figure S4: Dual dye control sample showing histograms of annexin FITC-A dye and propidium iodide dye which were used for setting the quadrants to analyse the early apoptotic cell populations in all the conditions (PI) (a) histogram of FITC-A, (b) histogram of PI; Figure S5: (a) gating of 2 h first replicate, (b) gating of 2 h second replicate, (c) gating of 2 h third replicate; Figure S6: (a) gating of 24 h continuous first replicate, (b) gating of 24 h continuous second replicate, (c) gating of 24 h continuous third replicate; Figure S7: (a) gating of 24 h non- continuous first replicate, (b) gating of 24 h non-continuous second replicate, (c) gating of 24 h non-continuous third replicate; Figure S8: (a) gating of 24 h control first replicate, (b) gating of 24 h control second replicate, (c) gating of 24 h control third replicate.
