# Supplementary material for: Kinetic Effects of Transferrin-Conjugated Gold Nanoparticles on the Antioxidant Glutathione-Thioredoxin Pathway
Source: Antioxidants (Basel). 2023 Aug 15;12(8):1617. doi: 10.3390/antiox12081617 (PMC10451790; doi:10.3390/antiox12081617)
Supplement: Supplementary file 1 [file antioxidants-12-01617-s001.zip › antioxidants-2455997-supplementary.pdf]

Supplementary Information

Article

Kinetic effects of transferrin-conjugated gold nanoparticles on the antioxidant glutathione-thioredoxin pathway

Sonia Sebastian [1 3], Manuela Klingler Hoffmann [2 3], Douglas Howard [1], Clifford Young [2 3], Jenni Washington [2 3], Harald Unterweger [4], Christoph Alexiou [4], Tyron Turnbull [1], Richard D'Andrea [5], Peter Hoffmann [2 3] and Ivan Kempson [1*]

1 Future Industries Institute, University of South Australia, 5095, South Australia
2 Mass Spectrometry & Proteomics Group, Clinical Health Sciences, University of South Australia, 5000 South Australia
3 Clinical Health Sciences, University of South Australia, 5000, South Australia
4 Department of Otorhinolaryngology, Head and Neck Surgery, Section of Experimental Oncology and Nanomedicine (SEON), Else Kröner-Fresenius-Stiftung Professorship, Universitätsklinikum Erlangen, Germany
5 Centre for Cancer Biology, University of South Australia, 5000 South Australia
* Correspondence: ivan.kempson@unisa.edu.au

Figure S1

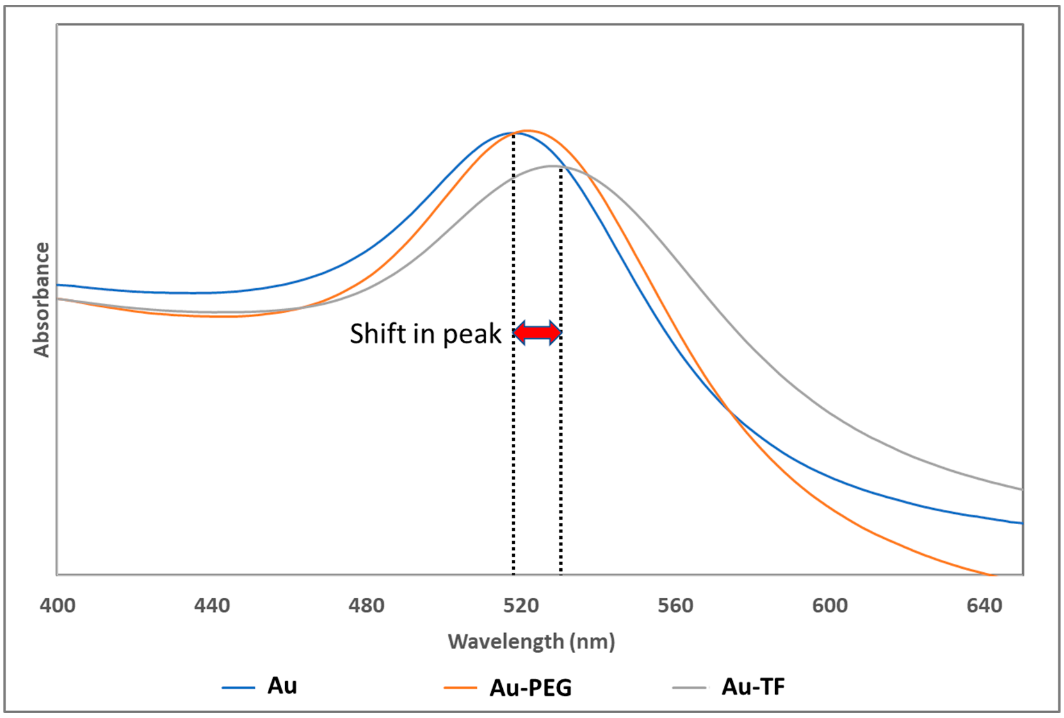

Figure S1 UV-VIS data showing shift in peak after PEG and transferrin conjugation onto Au nanoparticles.

Figure SI 2

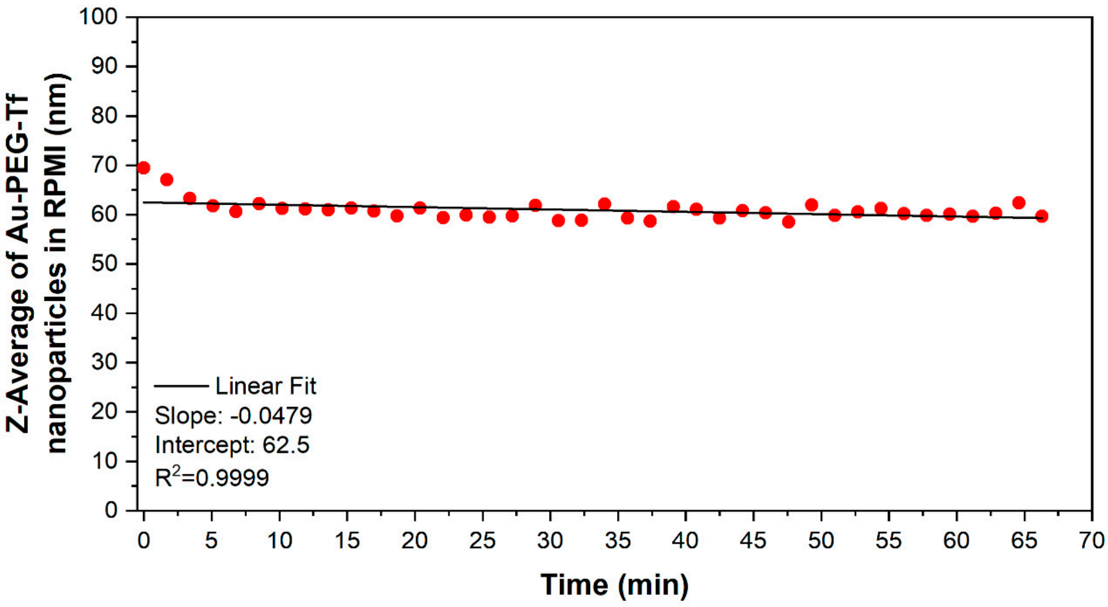

Figure S2 Stability measurement showing the Z-average of Au-Tf nanoparticles in RPMI (nm) with respect to time.

Figure SI 3

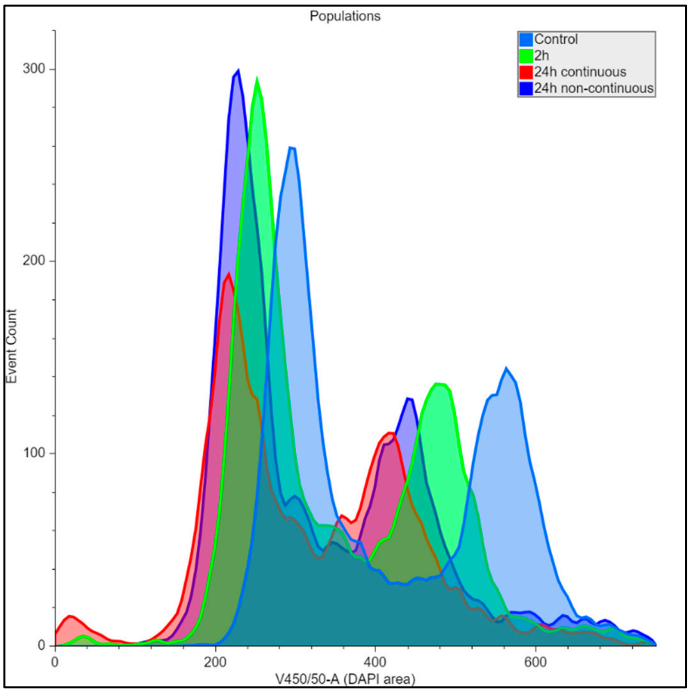

Figure S3 Cell cycle data of different treatment conditions with gold nanoparticles showing the sub G0/G1 peaks corresponding to apoptotic cell population.

Figure SI 4

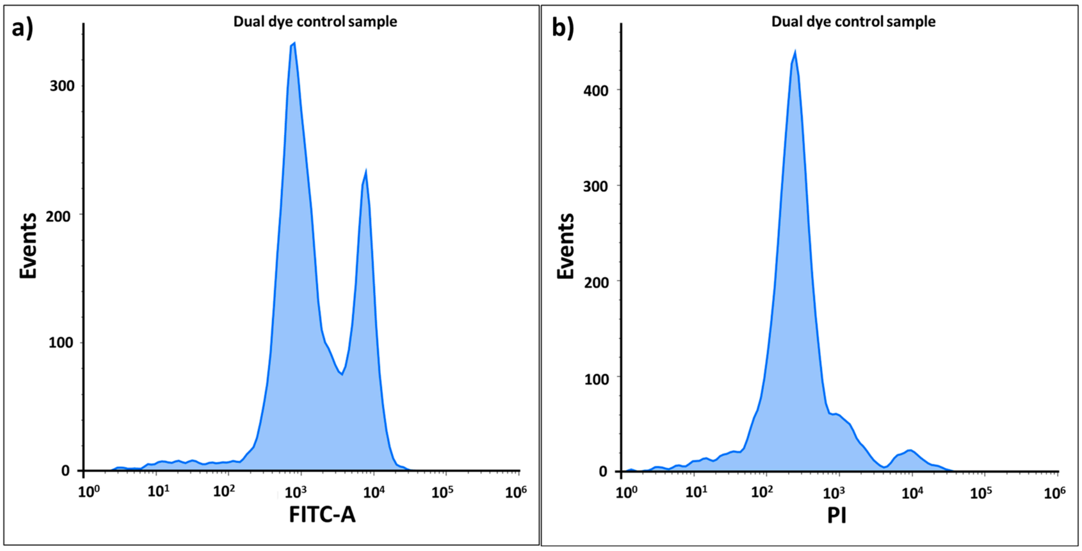

Figure S4 Dual dye control sample showing histograms of annexin FITC-A dye and propidium iodide dye which were used for setting the quadrants to analyse the early apoptotic cell populations in all the conditions (PI) (a) histogram of FITC-A, (b) histogram of PI

SI-1 Replicates for the early apoptotic assay

1. 2h exposure replicates

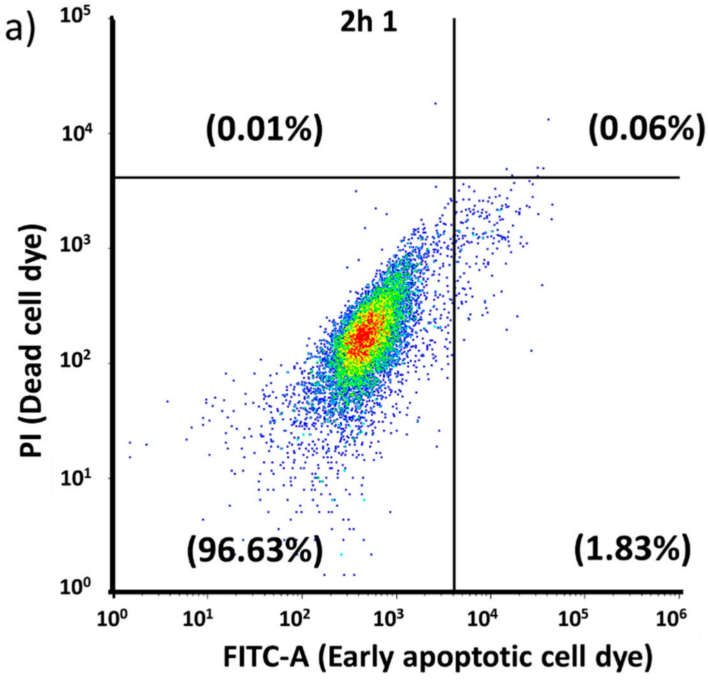

b)

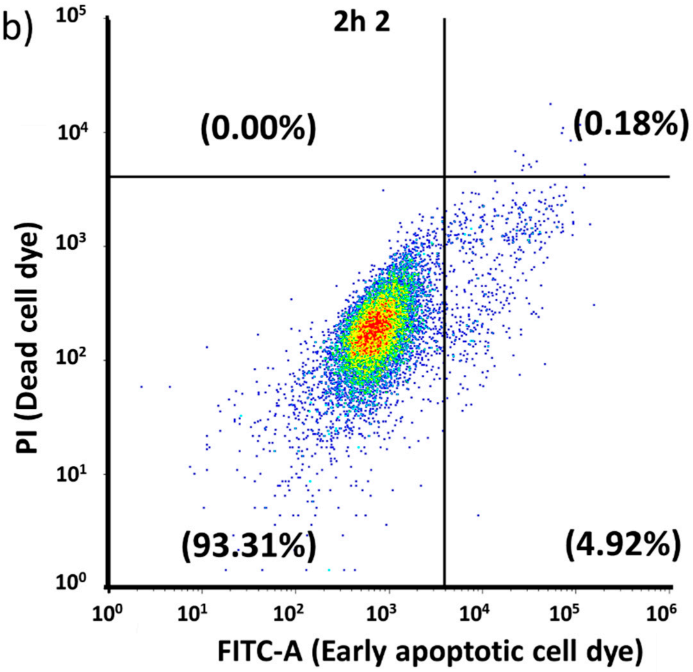

c)

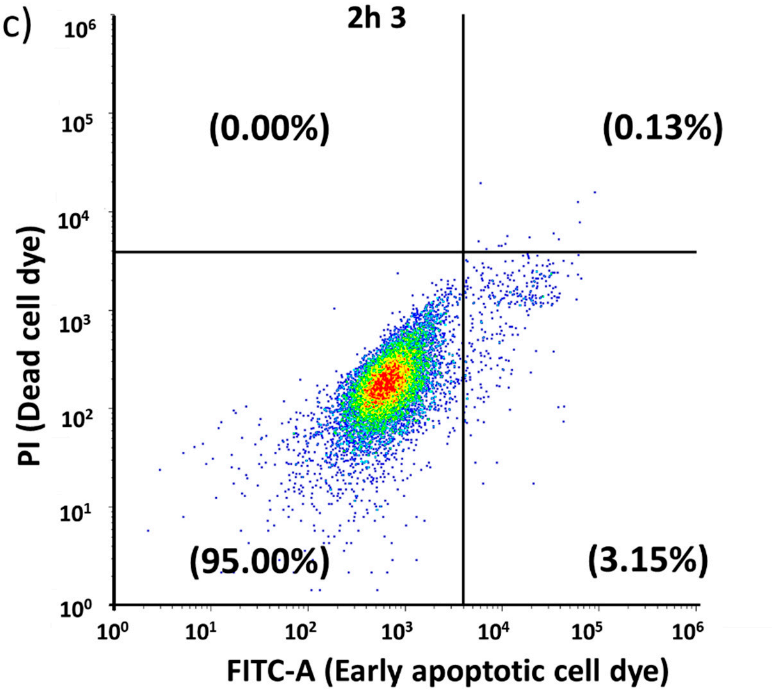

Figure S5 a) gating of 2 h first replicate, b) gating of 2 h second replicate, c) gating of 2 h third replicate

2. 24h continuous exposure replicates

a)

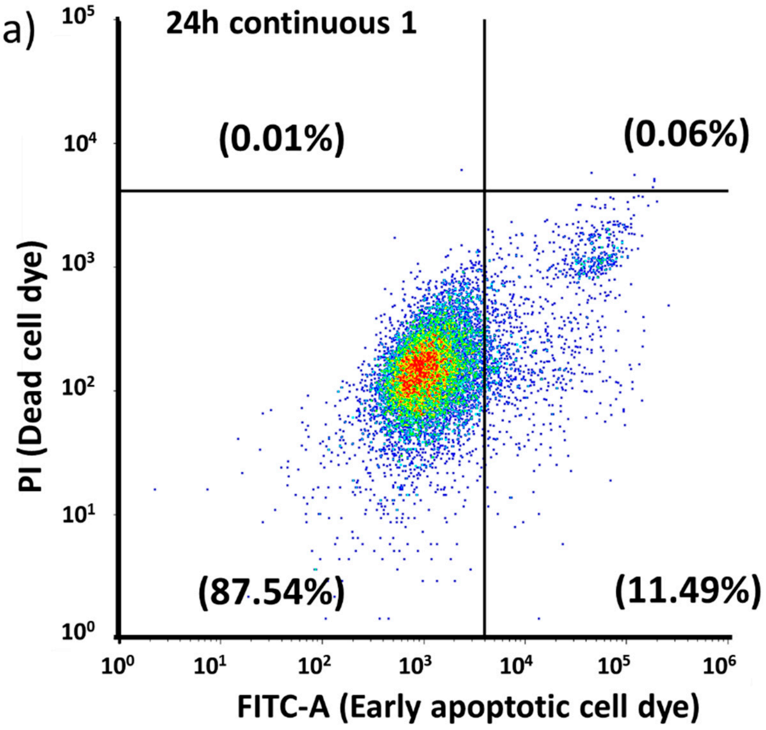

b)

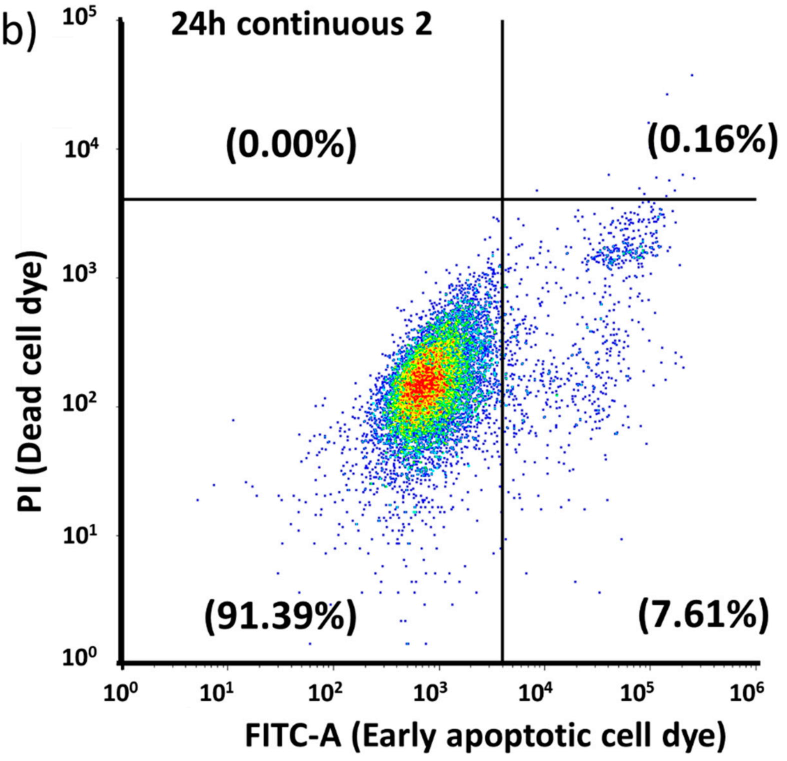

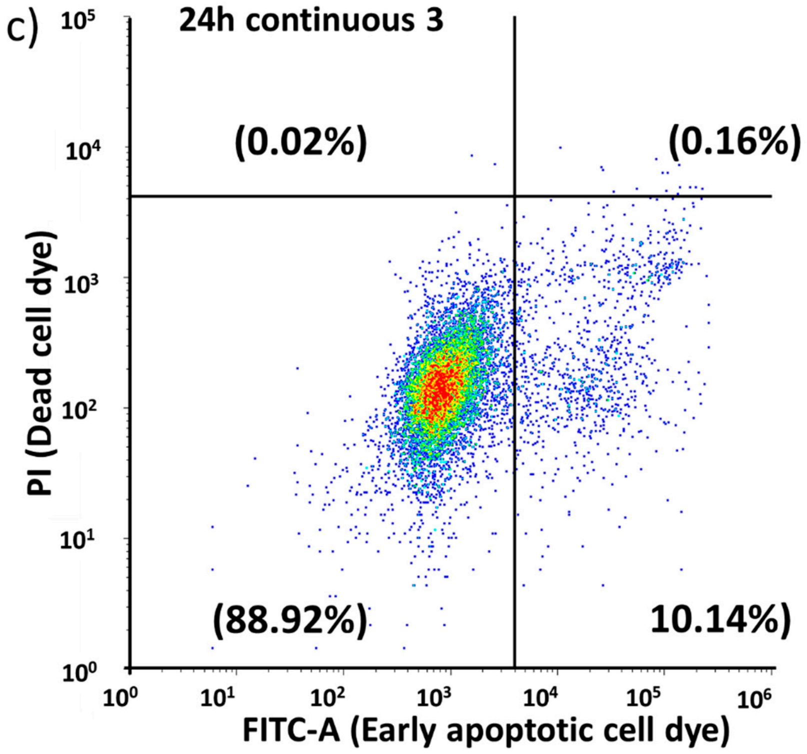

Figure S6 a) gating of 24 h continuous first replicate, b) gating of 24 h continuous second replicate, c) gating of 24 h continuous third replicate

3. 24 h non-continuous exposure replicates

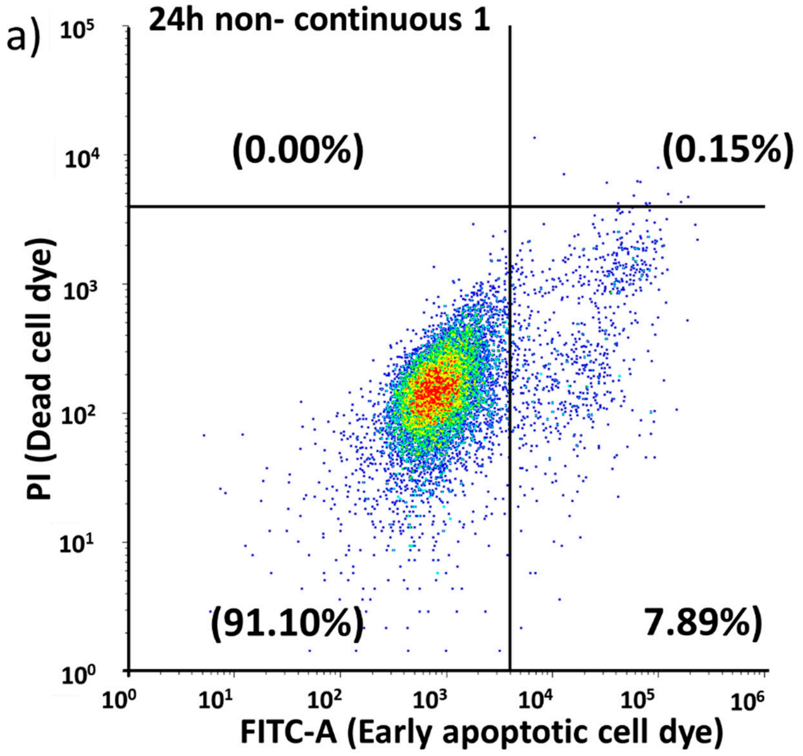

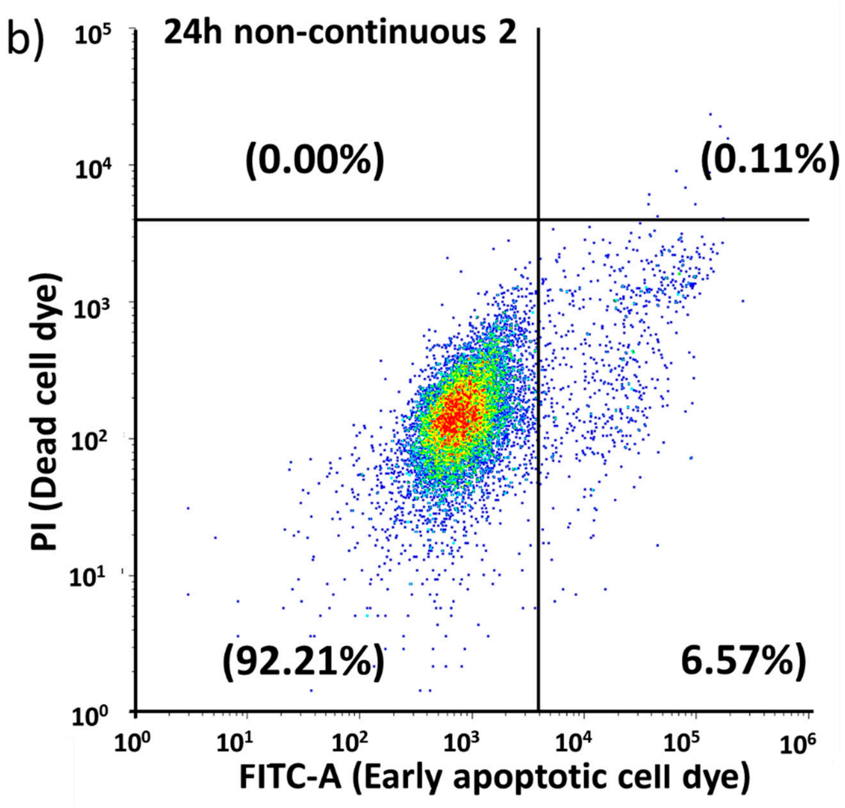

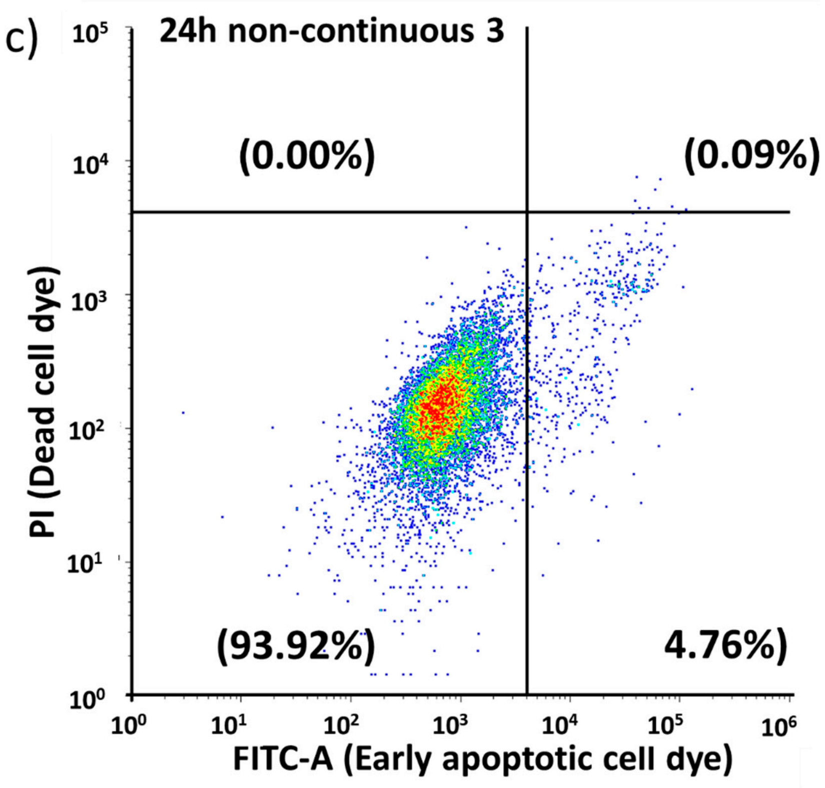

Figure S7 a) gating of 24 h non- continuous first replicate, b) gating of 24 h non-continuous second replicate, c) gating of 24 h non-continuous third replicate

4. 24 h control replicates

a)

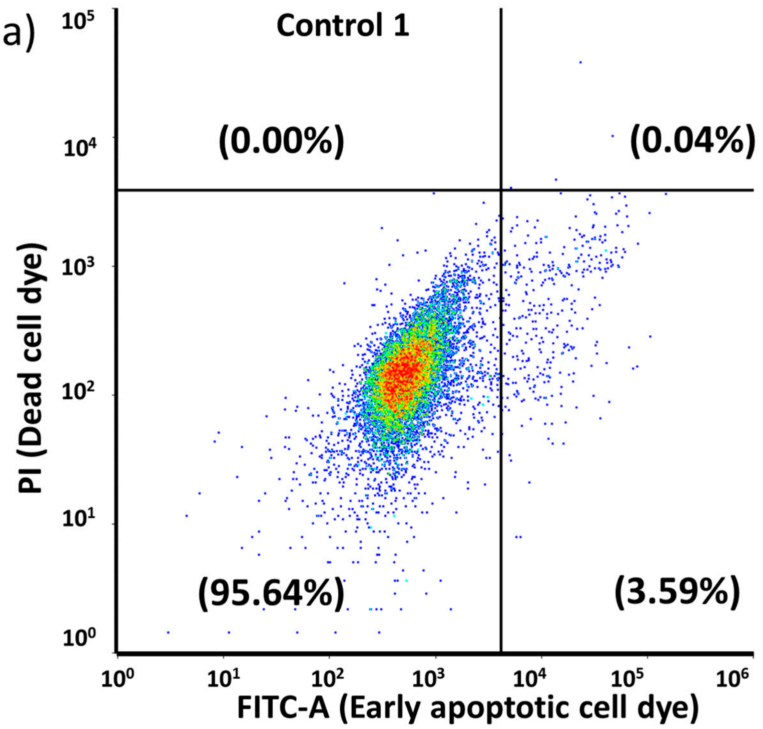

b)

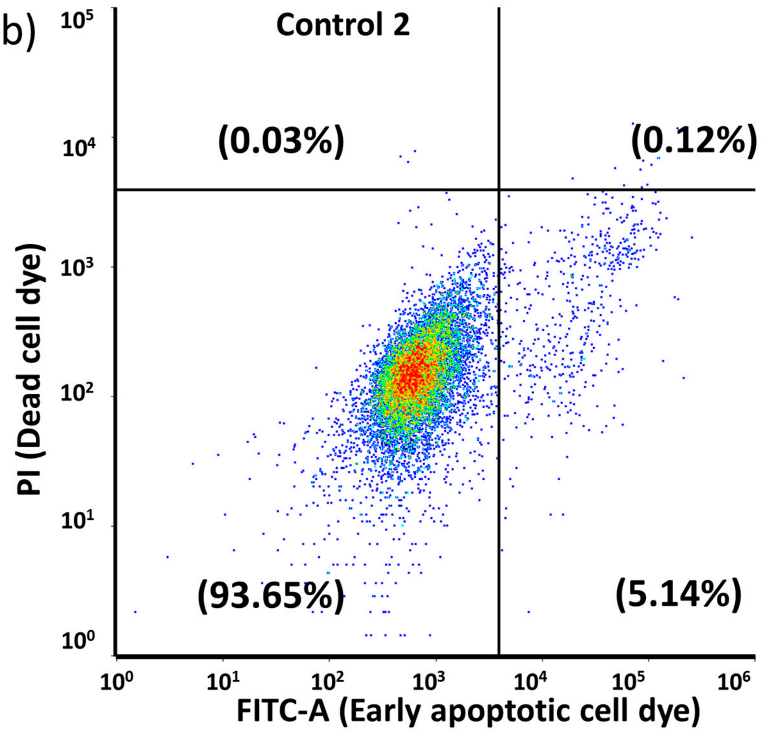

c)

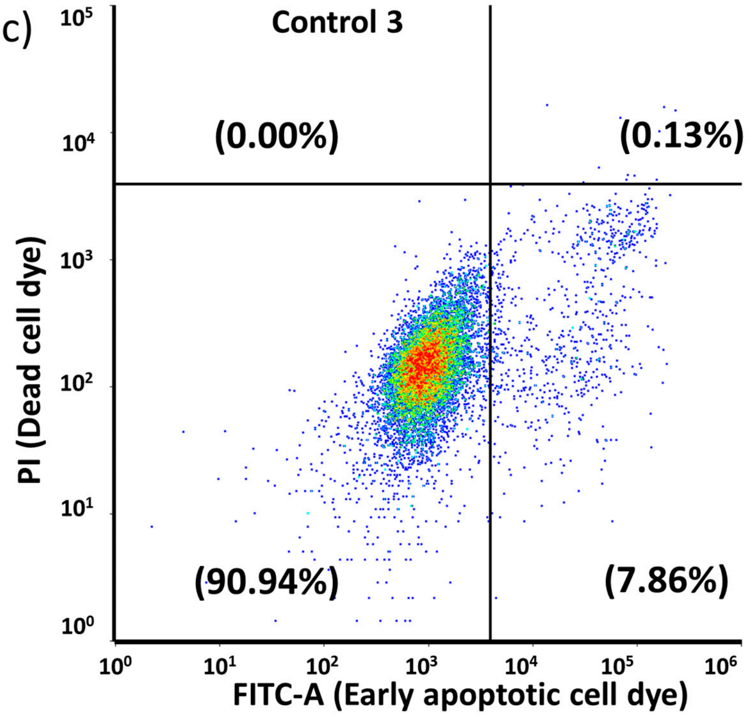

Figure S8 gating of 24 h control first replicate, b) gating of 24 h control second replicate, c) gating of 24 h control third replicate.
